# CD36 inhibition enhances the anti-proliferative effects of PI3K inhibitors in PTEN-loss anti-HER2 resistant breast cancer cells

**DOI:** 10.1186/s40170-025-00375-5

**Published:** 2025-02-07

**Authors:** You-Yu Liu, Wei-Lun Huang, Sin-Tian Wang, Hui-Ping Hsu, Tzu-Ching Kao, Wei-Pang Chung, Kung-Chia Young

**Affiliations:** 1 Institute of Basic Medical Sciences, College of Medicine, National Cheng Kung University, Tainan, 70101 Taiwan; 2 Center of Applied Nanomedicine, College of Medicine, National Cheng Kung University, Tainan, 70101 Taiwan; 3 Department of Medical Laboratory Science and Biotechnology, College of Medicine, National Cheng Kung University, No. 1 University Rd, Tainan, 70101 Taiwan; 4 Department of Surgery, College of Medicine, National Cheng Kung University Hospital, National Cheng Kung University, Tainan, 70101 Taiwan; 5 Department of Oncology, College of Medicine, National Cheng Kung University Hospital, National Cheng Kung University, No. 1 University Rd, Tainan, 70101 Taiwan

**Keywords:** anti-HER2 resistant breast cancer, PI3K inhibitors, PTEN-loss, CD36 fatty acids transporter, Fatty acids metabolism

## Abstract

**Background:**

HER2-positive patients comprise approximately 20% of breast cancer cases, with HER2-targeted therapy significantly improving progression-free and overall survival. However, subsequent reprogramed tumor progression due to PI3K signaling pathway activation by *PIK3CA* mutations and/or PTEN-loss cause anti-HER2 resistance. Previously, alpha isoform-specific PI3K inhibitors were shown to potentiate HER2-targeted therapy in breast cancer cells carrying PI3K pathway alterations with less potent effects on PTEN-loss than *PIK3CA*-mutant cells. Therefore, seeking for alternative combination therapy needs urgent attentions in PTEN-loss anti-HER2 resistant breast cancer.

**Methods:**

Since remodeling of fatty acid (FA) metabolism might contribute to HER-positive breast cancer and is triggered by the PI3K signal pathway, herein, we examined the effects of the inhibition of endogenous FA conversion, SCD-1 or exogenous FA transport, CD36, in combination with PI3K inhibitors (alpelisib and inavolisib) in anti-HER2 resistant PTEN-loss breast cancer cells.

**Results:**

The activated HER2/PI3K/AKT/mTOR signaling pathway positively correlated with SCD-1 and CD36 expression in PTEN-loss breast cancer cells. PI3K inhibition downregulated SCD-1, and accordingly, the addition of the SCD-1 inhibitor did not augment the antiproliferative effects of the PI3K inhibitors. CD36 was upregulated by blocking the PI3K signal pathway or limited serum supplementation, indicating that suppressing CD36 may decrease the excess transport of exogenous FA. The addition of the CD36 inhibitor synergistically enhanced the anti-proliferative effects of the PI3K inhibitors.

**Conclusion:**

Simultaneously targeting the PI3K signaling pathway and exogenous FA uptake could potentially be advantageous for patients with PTEN-loss anti-HER2 resistant breast cancer.

**Supplementary Information:**

The online version contains supplementary material available at 10.1186/s40170-025-00375-5.

## Introduction

Human epidermal growth factor receptor 2 (HER2) is an important regulator involved in breast cancer progression. Around 20% of breast cancer patients are the HER2-positive subtype [1] and have a high risk of relapse, metastasis, and poor prognosis [2]. The HER2-targeted therapy drugs, including monoclonal antibody-based regimens, trastuzumab, and pertuzumab, have substantially improved progression-free and overall survival in patients with HER2-positive breast cancer [3–5]. However, cancer recurrence due to resistance to the anti-HER2 treatments can occur so there is still an urgent need for various therapeutic strategies.

HER2 dimerization and phosphorylation initiate signaling that leads to activation of downstream proteins including phosphoinositide 3-kinases (PI3K), protein kinase B (PKB, also known as AKT), and mammalian target of rapamycin (mTOR), thereby transducing signals for cell proliferation, cell survival, and angiogenesis in various types of cancer, including breast cancer [6, 7]. The genetic mutation of *PIK3CA* at the p110α subunit cause unregulated catalytic activity to phosphorylate phosphatidylinositol 4,5-bisphosphate (PIP2) which is converted to phosphatidylinositol-3, 4, 5-triphosphate (PIP3), facilitating AKT and sequential mTOR activation by phosphorylation at Thr308 or Ser473 [6–9]. In the HER2/PI3K/AKT/mTOR regulatory axis, the phosphatase and tensin homolog (PTEN) protein can dephosphorylate PIP3 to PIP2 and function as a negative regulator [9]. In HER2-positive breast cancer cells, *PIK3CA* mutations and PTEN loss cause constitutive activation of PI3K/AKT/mTOR signals leading to oncogenesis as well as anti-HER2 resistance [10, 11]. Subsequently, alpha isoform-specific PI3K inhibitors have been developed as targeted therapy drugs. Among them, alpelisib is now clinically available to treat hormone receptor-positive/HER2-negative, *PIK3CA*-mutated breast cancer, whereas inavolisib, more potent than alpelisib in vitro, is under investigation in phase III clinical trials [12, 13]. Furthermore, blocking PI3K is also proposed to treat HER2-positive breast cancer [14] and exert synergistic effects in combination with trastuzumab for the treatment of anti-HER2-resistant breast cancer with either a *PIK3CA* mutation or loss of PTEN expression, where the therapeutic effectiveness is higher in *PIK3CA*-mutant cells [15].

Metabolic remodeling of cancer cells with tumor microenvironment interactions promotes survival, proliferation, and metastasis [16]. The surrounding cancer-associated adipocytes contribute lipid energy supply in the context of signal secretion to support breast cancer invasion and progression [17–19]. A cancer hallmark is the lipid reprogramming of fatty acid (FA) metabolism through endogenous lipogenesis and exogenous uptake pathways [20, 21]. PI3K activation transduces signals to sterol regulatory element-binding protein 1 (SREBP-1) and up-regulates stearoyl-CoA desaturase-1 (SCD-1) expression to facilitate the endogenous production of monounsaturated FAs (MUFAs) [22–26] which positively regulate AKT phosphorylation [27, 28]. Cluster of designation 36 (CD36) is considered the predominant membrane protein facilitating FA transport [29] and is overexpressed in HER2-positive breast cancer and associated with enhanced cell proliferation and migration [30].

Since lipid metabolic remodeling as an emerging mechanism participate in resistance to kinase inhibitors [31], we hypothesize that the inhibition of SCD-1 or CD36 might potentiate the effects of current PI3K-targeting agents in breast cancer with resistance to anti-HER2 drugs. Herein, our study indicates that simultaneous inhibition of PI3K/AKT/mTOR signaling and exogenous FAs uptake using a combination of PI3K and CD36 inhibitors might reduce cell proliferation synergistically in anti-HER2 resistant breast cancer with PTEN-loss.

## Methods

### Cell culture

The three breast cancer cell lines used were purchased from the American Tissue Culture Collection (ATCC) and maintained at 37 °C in ATCC-recommended growth media (GM) with 10% fetal bovine serum (FBS) as follows: Dulbecco’s Modified Eagle medium for MDA-MB-231 cells, MyCoy’s 5A medium for SK-BR-3 cells, and RPMI-1640 medium (Thermo Fisher Scientific, MA, USA) for HCC1569 cells. The cells were seeded (7.5 × 10^5^*per* well) in 10-cm culture dishes in serum-free medium (SFM) and incubated overnight at 37 °C and 5% CO_2_. The GM was replaced with 1% SFM and the cell morphology was observed daily by microscopy.

### Inhibitor treatment

The cells were seeded (1 × 10^4^*per* well) in 96-well culture plates and incubated overnight at 37 °C and 5% CO_2_ before treatment with the inhibitors alpelisib, inavolisib, A939572, and sulfosuccinimidyl oleate (SSO) (MedChemExpress, NJ, USA) for 72 h. All the drugs were dissolved using dimethyl sulfoxide as vesicle (Merck KGaA, DA, Germany). The cell lysates were then harvested for further analysis. Combination therapy was arranged to involve the strategy of using low concentrations that induce individually ~ 20–40% cytotoxicity of two or multiple drugs, as the drug doses and treatment duration were justified according to our previous study [15].

### Cell proliferation

The cells were grown (1 × 10^4^*per* well) in 96-well plates with and without the indicated treatment before cell proliferation was evaluated by incubation with 20 µL of WST-1 (Takara, Kyoto, Japan) reagent for 1 h at 37 °C. The absorbance was measured at 450 nm with an ELISA reader (Varioskan; Thermo Fisher Scientific, MA, USA). The cell doubling time was calculated as follows: doubling time = (log 2) × (t1 - t0) / (log X2 - log X1), where X represents cell number, and t represents the number of days after treatment.

### Western blotting

The whole cell lysates were separated by SDS-PAGE before transfer to polyvinylidene fluoride membranes (Merck KGaA, DA, Germany). The membranes were incubated overnight with specific primary antibodies to HER2, β-actin (Merck KGaA, DA, Germany), PI3K p110α, PTEN, pS473-AKT, AKT, pS244/S240-S6, S6, pS2448-mTOR, mTOR (Cell Signaling Technology, MA, USA), CD36 (Proteintech, IL, USA), SREBP-1 (Novus Biologicals, CO, USA) or SCD-1 (Abcam, Cambridge, UK). The membranes were then incubated with anti-mouse and anti-rabbit (Jackson ImmunoResearch, PA, USA) secondary antibodies, and the protein bands were detected with an enhanced chemiluminescence kit (Thermo Fisher Scientific, MA, USA). The intensity of each protein band was quantified using ImageJ (National Institutes of Health, USA), normalized to β-actin, and expressed relative to the control.

### Combination index (CI)

The anti-proliferative results of the individual treatment and their co-treatment were analyzed to determine CI values by isobologram using CompuSyn software [32]. The synergy was defined by CI < 1, additive effect by CI = 1 and antagonism by CI > 1.

### Statistical analysis

All experiments were repeated 3–6 times and the results are presented as mean ± standard error. The differences between groups were determined by unpaired Student t-tests in GraphPad Prism. A P-value < 0.05 was considered statistically significant (*p ≤ 0.05, **p ≤ 0.01, and ***p < 0.001).

## Results

### Dual activation of SCD-1 and CD36 in HER2-positive breast cancer

**Fig. 1:**
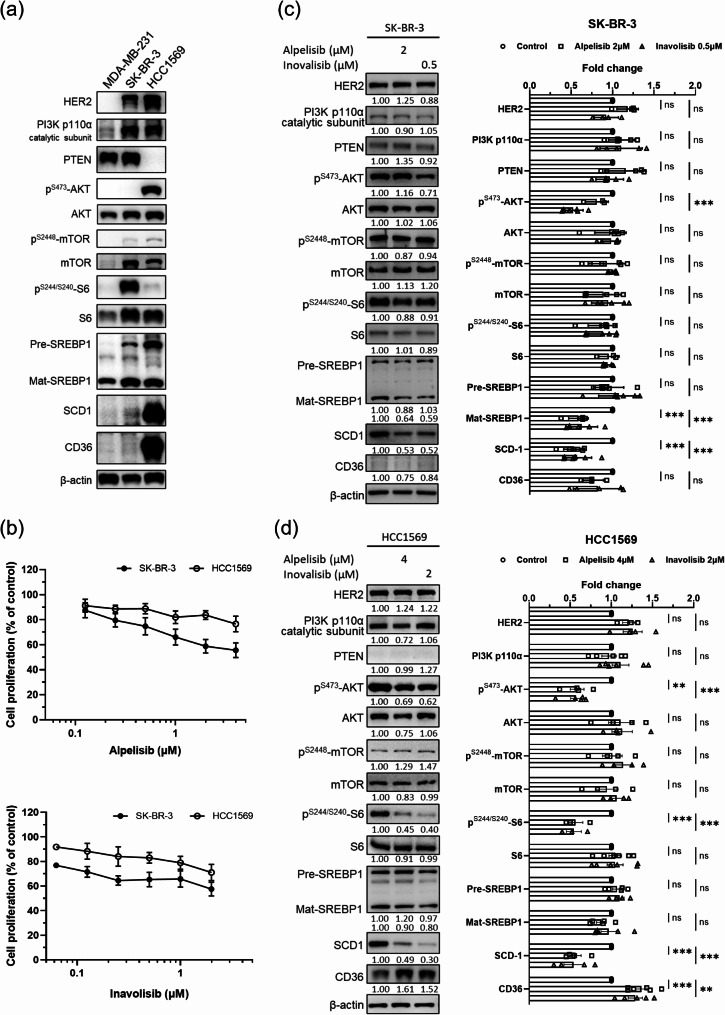
Modulation of activated SCD-1 and CD36 by PI3K inhibitors in HER2-positive breast cancer cells. (**a**) The basal protein levels of SCD-1 and CD36 were assessed by western blotting in MDA-MB-231 (HER2-negative), SK-BR-3 (HER2-positive), and HCC1569 (HER2-positive/PTEN-loss) breast cancer cells. Pre: precursor; Mat, mature. (**b**) The effects of PI3K inhibitors alpelisib (upper panel) and inavolisib (bottom panel) on the proliferation of SK-BR-3 and HCC1569 cells. The cell numbers were assessed by the WST-1 assay and the results are presented as a percentage of the untreated control (mean ± SEM). (**c**, **d**) The modulated protein levels of SCD-1 and CD36 were assessed by western blotting in SK-BR-3 and HCC1569 cells treated with PI3K inhibitors for 24 h. The protein expression is expressed relative to the untreated control (mean ± SEM), **P < 0.01 and ***P < 0.001

MDA-MB-231 was used as the cell model for HER2-negative breast cancer, SK-BR-3 for HER2-positive anti-HER2 sensitive breast cancer, and HCC1569 cells for anti-HER2 resistant with PTEN-loss (Fig. 1a). The correlations between HER2/PI3K/AKT/mTOR and lipid metabolism-related proteins in Fig. 1a show that SK-BR-3 and HCC1569 cells expressed more SREBP, SCD-1 and CD36 compared to MDA-MB-231 cells. The upregulated expression was more profound in HCC1569 cells which were constitutively activated due to the loss of PTEN. Based on the results, it is appropriate to hypothesize that the de novo lipogenesis and exogenous FA uptake might be upregulated in HER2-positive breast cancer cells by PI3K/AKT/mTOR activation and, therefore, to evaluate therapeutics targeting PI3K in combination with SCD-1 or CD36 inhibitors in HER2-positive breast cancers.

### PI3K inhibitors decreased SCD-1 expression and induced CD36 expression in HER2-positive breast cancer cells

The PI3K inhibitor alpelisib has been approved for the treatment of hormone receptor-positive/HER2-negative breast cancer, while inavolisib is under investigation. In addition, synergistic effects have been shown between these PI3K inhibitors and trastuzumab in anti-HER2-resistant breast cancers [15]. In this study, the PI3K inhibitors reduced the cell proliferation of two HER2-positive breast cancer cell lines, where SK-BR-3 exhibited more susceptibility to alpelisib or inavolisib than HCC1569 (Fig. 1b). In addition, when the PI3K/AKT/mTOR signaling was blocked by alpelisib or inavolisib, the *de novo* lipogenesis SCD-1 protein was reduced substantially in both SK-BR-3 and HCC1569 cells, and the SREBP1 protein was decreased in SK-BR-3 (Fig. 1c and d). These results support the potential role of the PI3K/AKT/mTOR signaling in controlling *de novo* lipogenesis in HER2-positive breast cancer cells and show that PI3K inhibitors can decrease MUFAs production by inhibiting SCD-1 expression. Simultaneously, PI3K inhibitors upregulated CD36 in HCC1569 cells (Fig. 1d).

### Modulation of de novo lipogenesis SCD-1 in combination with PI3K inhibitors in anti-HER2 resistant breast cancer with PTEN-loss

**Fig. 2:**
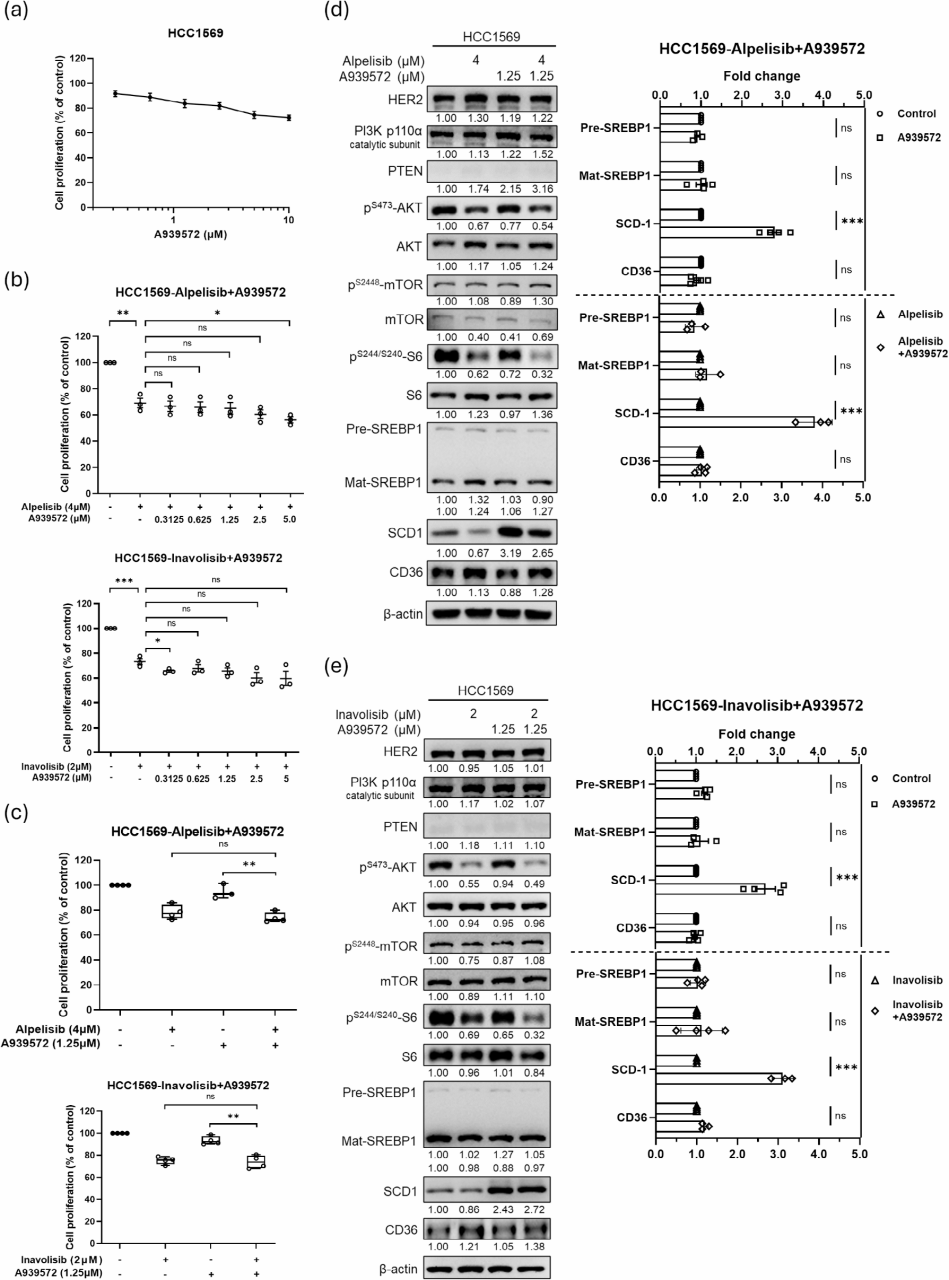
The effects of the combination of SCD-1 and PI3K inhibitors in HER2-positive PTEN-loss breast cancer cells. The effects on the proliferation of HCC1569 cells treated with the SCD-1 inhibitor (**a**), and alpelisib (**b**) or inavolisib (**c**). The cell numbers were assessed using the WST-1 assay and presented as a percentage of the untreated control (mean ± SEM). The modulated protein levels of SCD1 and CD36 were evaluated by western blotting analysis in HCC1569 cells treated with A939572 plus alpelisib (**d**) or inavolisib (**e**) for 24 h. The protein expression is expressed relative to the untreated control (mean ± SEM), *P < 0.05, **P < 0.01 and ***P < 0.001

Next, we focused on investigating the potential combined effects of PI3K and lipid metabolic inhibitors for treating HER2-resistant breast cancers with PTEN-loss using the HCC1569 cell model. Treatment with the SCD-1 inhibitor (A939572) dose-dependently reduced HCC1569 cell proliferation (Fig. 2a) but co-treatment with alpelisib or inavolisib showed limited additive effects on proliferation (Figs. S2, 2b and 2c). SCD-1 protein expression significantly increased 3-fold with the treatment of A939572 alone compared to the untreated control (Fig. 2d and e), as well as 3-fold higher with the co-treatment of PI3K and SCD-1 inhibitors than with alpelisib or inavolisib alone (Fig. 2d and e).

### Blocking exogenous lipid uptake by serum depletion inhibited cell proliferation and up-regulated CD36 expression in anti-HER2 resistant breast cancer with PTEN-loss

**Fig. 3:**
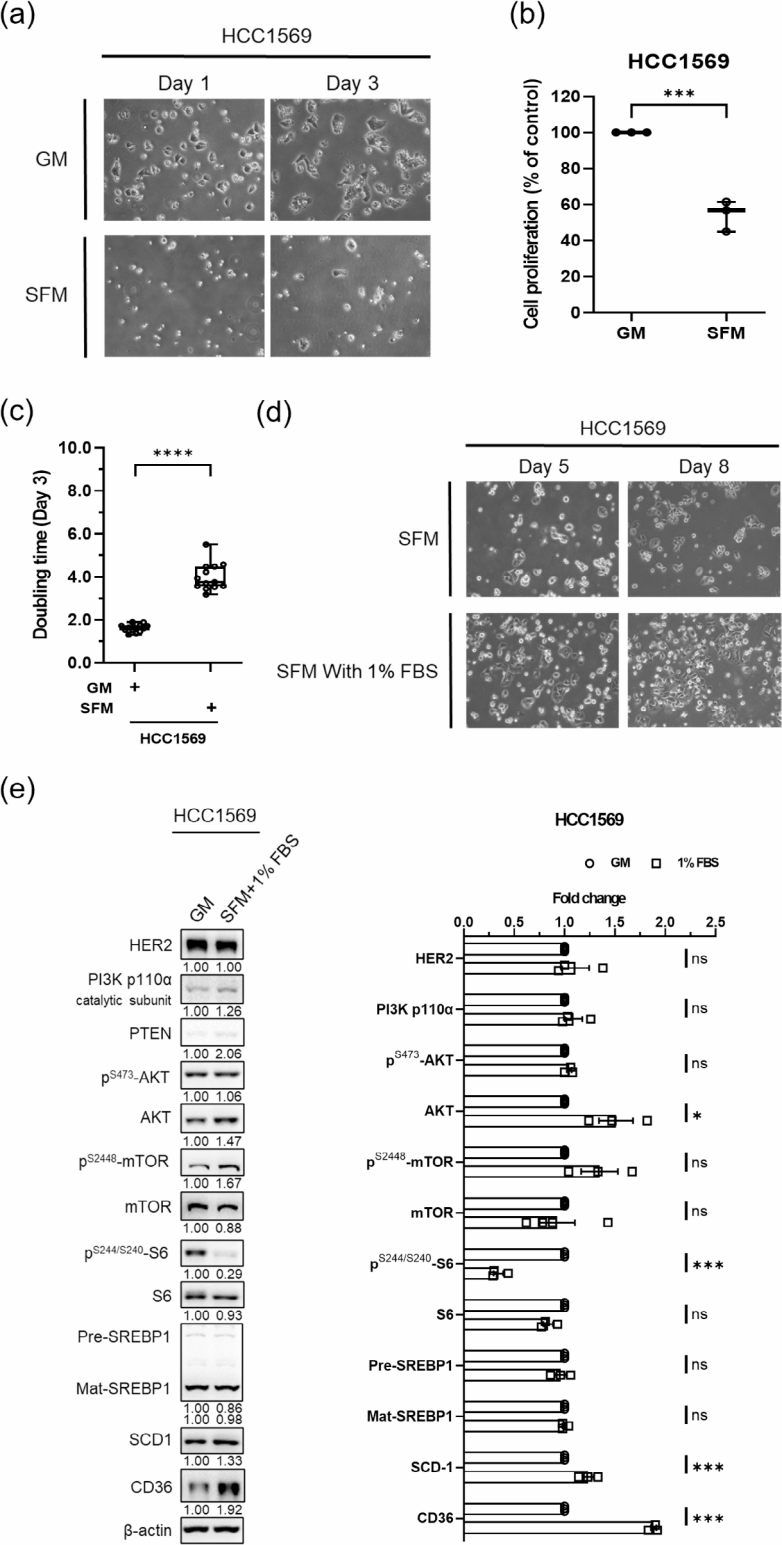
Serum-deficient medium inhibited cell proliferation and up-regulated CD36 in HER2-positive PTEN-loss breast cancer cells. The HCC1569 cells were incubated in GM or SFM and (**a**) the cell morphology was visualized on day 1 and day 3, (**b**) the cell number was evaluated using the WST-1 assay at 72 h and (**c**) the doubling time was calculated. The results in b and c were presented as the box and whiskers with minimum to maximum. The HCC1569 cells were incubated in SFM for 24 h and then the media were replaced by SFM medium with or without 1% serum and (**d**) the cell morphology was visualized on day 5 and day 8, and (**e**) the modulated protein levels of SCD-1 and CD36 were evaluated by western blotting at 24 h. The protein expression is presented relative to the control (mean ± SEM), *P < 0.05, **P < 0.01, ***P < 0.001 and ****P < 0.0001

In addition to endogenous *de novo* lipogenesis, intracellular FAs can be obtained by uptake from exogenous sources, such as serum in the medium. When HCC1569 cells were cultivated in SFM, cell proliferation was significantly reduced (Fig. 3a) to 60% of the GM control after 3 days (Fig. 3b), whereas the doubling time was prolonged from 1.62 ± 0.05 to 4.00 ± 0.18 days (Fig. 3c). Furthermore, the morphology and proliferation of HCC1569 cells were partially restored as the addition of 1% serum to the medium (Fig. 3d). As compared to the GM (10% FBS), the cells cultured in SFM with supplement of 1% FBS (serum reduction) for 24 h exhibited minimal changes in HER2, PI3K, phospho-AKT, phospho-mTOR and SREBP1, but significantly upregulated SCD-1 (~ 30%) and CD36 (~ 90%) (Fig. 3e). These results suggest that blocking exogenous lipid uptake by serum depletion might inhibit cell proliferation in the context of up-regulation of CD36 which is the feedback response due to serum reduction. Taken together, HCC1569 cell proliferation might be accelerated with serum providing exogenous FAs, which are delivered by surface CD36.

### Modulation of FA uptake through CD36 in combination with PI3K inhibitors in anti-HER2 resistant breast cancer with PTEN-loss

**Fig. 4:**
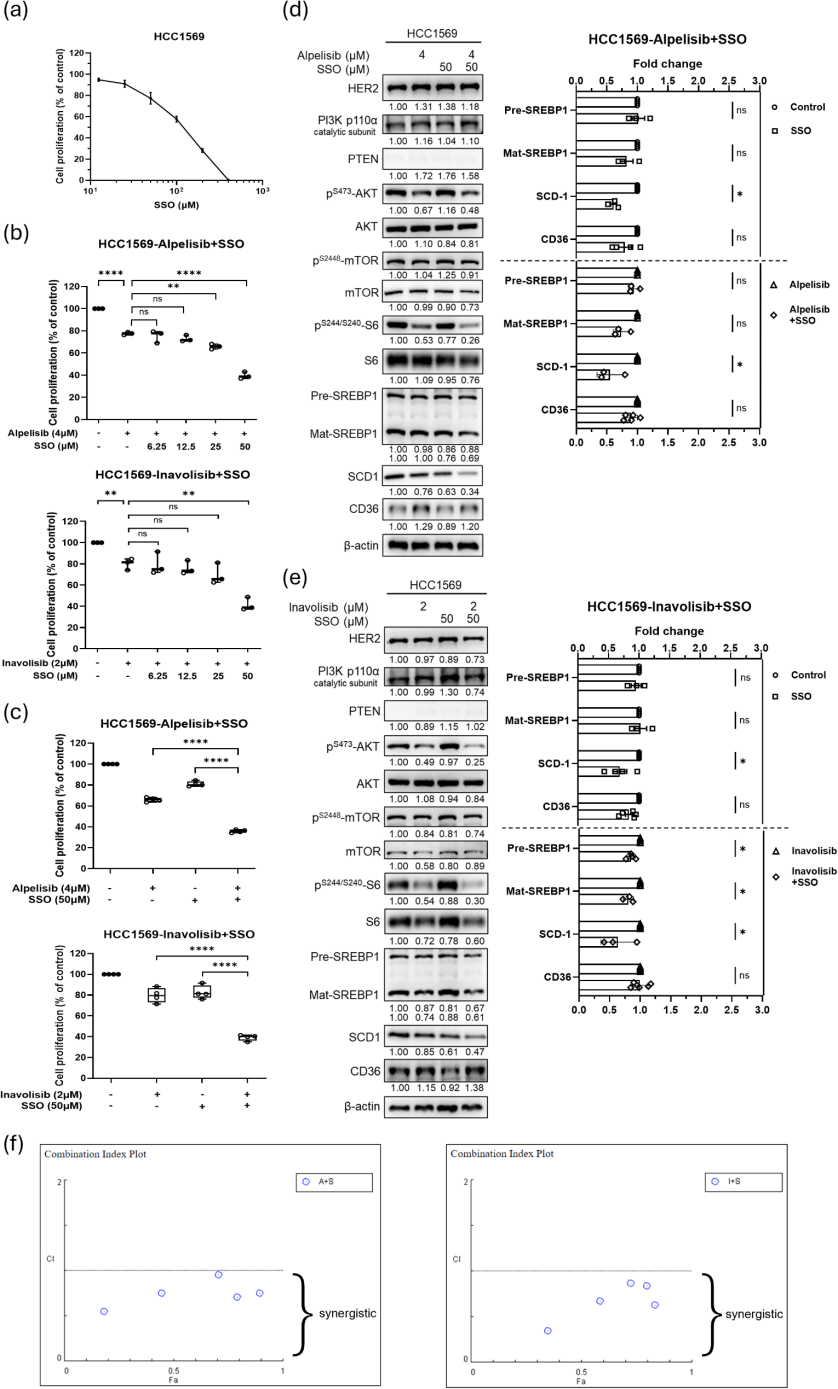
The effects of the combination of CD36 and PI3K inhibitors in HER2-positive PTEN-loss breast cancer cells. (**a**) The effects on the proliferation of HCC1569 cells treated with the CD36 inhibitor. The cell numbers were assessed in SSO-treated cells for 72 h using the WST-1 assay and presented as a percentage of the untreated control (mean ± SEM). The effects on cell proliferation of HCC1569 cells treated with the CD36 inhibitor in combination with alpelisib (**b**) or inavolisib (**c**) for 72 h. The cell numbers were assessed by the WST-1 assay and presented as a percentage of the untreated control (mean ± SEM). (**d**, **e**) The modulated protein levels of SCD-1 and CD36 were evaluated by western blotting in HCC1569 cells treated with SSO plus alpelisib (**d**) or inavolisib (**e**) for 24 h. The protein expression is relative to the untreated control (mean ± SEM), *P < 0.05, **P < 0.01, ***P < 0.001 and ****P < 0.0001. (f) Isobologram analysis for the combined treatments of alpelisib (left) or inavolisib (right) with SSO in HCC1569 cells for 72 h. CI < 1 indicated synergistic effect. A: alpelisib; I: inavolisib; S: SSO

Since PI3K inhibitors (Fig. 1d) and serum reduction (Fig. 3e) induced substantially increasing of CD36 expression and blocking exogenous lipid uptake by serum depletion inhibited cell proliferation (Fig. 3a-c), manipulation of CD36 might affect the proliferation of HCC1569 cells. Treatment with the CD36 inhibitor (SSO) reduced the cell number to 60% (Fig. 4a), suggesting that the role of CD36 in the transport of exogenous FAs might be associated with HCC1569 cell proliferation. To confirm the anti-proliferative effects regarding WST-1 assay for accurately assessing cell number in the presence of therapeutic treatment which might alter cellular metabolism, we performed the experiments using trypan blue exclusion assay in parallel. The results exhibited similar inhibitory patterns by the two assays with the treatments of alpelisib (4 µM), inavolisib (2 µM), A939572 (1.25 µM), and SSO (50 µM) in HCC1569 cells at 72 h (Fig. S1). Therefore, we hypothesized that inhibition of the FA transporter might enhance the effect of PI3K inhibition in anti-HER2-resistant breast cancer with PTEN-loss.

Co-treatment with 50 µM SSO significantly enhanced the anti-proliferative effects of alpelisib and inavolisib in HCC1569 cells (Fig. 4b and c). Notably, combination of either alpelisib (Fig. 4f, left) or inavolisib (Fig. 4f, right) with SSO in HCC1569 cells showed synergistic effects of combined PI3K and CD36 inhibition. Furthermore, the combination of alpelisib or inavolisib and SSO decreased SCD-1 expression compared to the corresponding controls without SSO (Figs. S3, 4d and 4e). This suggests that the dual inhibition of exogenous FA uptake and endogenous SCD-1-mediated *de novo* lipogenesis using CD36 and PI3K inhibitors might be a viable therapeutic approach in anti-HER2 resistant breast cancer with PTEN-loss. To confirm the impact of combined inhibitions of PI3K and FA uptake is more pronounced in PTEN-deficient breast cancers. HCC1954 (*PIK3CA* mutant) and SK-BR-3 cells were utilized to test the treatment effects on breast cancers with intact PTEN. The results showed that co-treatment with SSO might exhibit minimal enhanced anti-proliferative effects of alpelisib and inavolisib in HCC1954 (Fig. S4a) and SK-BR-3 (Fig. S4b) cells as compared to those in HCC1569 cells (Fig. 4c). These results suggest that combined inhibition of PI3K and FA uptake is more specific synergism in HER2-positive breast cancer with PTEN loss.

To evaluate the effects of PI3K and CD36 inhibition, intracellular fatty acid levels were quantified in HCC1569 cells following treatment with PI3K inhibitors and the CD36 inhibitor. The results showed an approximately 20% increase in intracellular FA levels with PI3K inhibition alone, while no significant changes were observed with either SSO treatment alone or the combination of alpelisib and SSO. (Fig. S5a). To evaluate the effects of PI3K and CD36 inhibition on mitochondrial FA utilization, the expression levels of carnitine palmitoyltransferase 1 A (CPT1A), the rate-limiting enzyme in mitochondrial FA oxidation, were measured. The results showed no significant changes with PI3K inhibition alone, a reduction of approximately 25% with SSO treatment alone, and a similar reduction with the combined treatment of PI3K inhibitors and SSO. (Fig. S5b). These results suggest that the PI3K and CD36 pathways interact to regulate intracellular FA availability in HER2-positive breast cancer cells, with the effects of CD36 inhibition potentially dominating over those of PI3K inhibition. Consequently, the addition of CD36 inhibitor could reduce cellular FA uptake and mitochondrial β-oxidation, thereby limiting energy production and enhancing the anti-proliferative effects of PI3K inhibition.

## Discussion

Lipid metabolism-related proteins are therapeutic targets for cancer treatment, for example, targeting the *de novo* lipogenesis using SCD-1 inhibitors [33]. The SCD-1 inhibitor, A939572, was included in a preclinical trial of glioblastoma and renal cell carcinoma [28]. Blocking lipid uptake by CD36 inhibition is a potential therapy for prostate cancer [34]. Moreover, both *de novo* FA lipogenesis and exogenous FA uptake pathways contribute to the pathogenesis of HER2-positive breast cancer [22–26]. Therefore, we proposed that the inhibition of endogenous FA lipogenesis and/or exogenous FAs uptake might be promising to enhance the effect of agents targeting PI3K in breast cancer with resistance to anti-HER2 drugs.

**Fig. 5:**
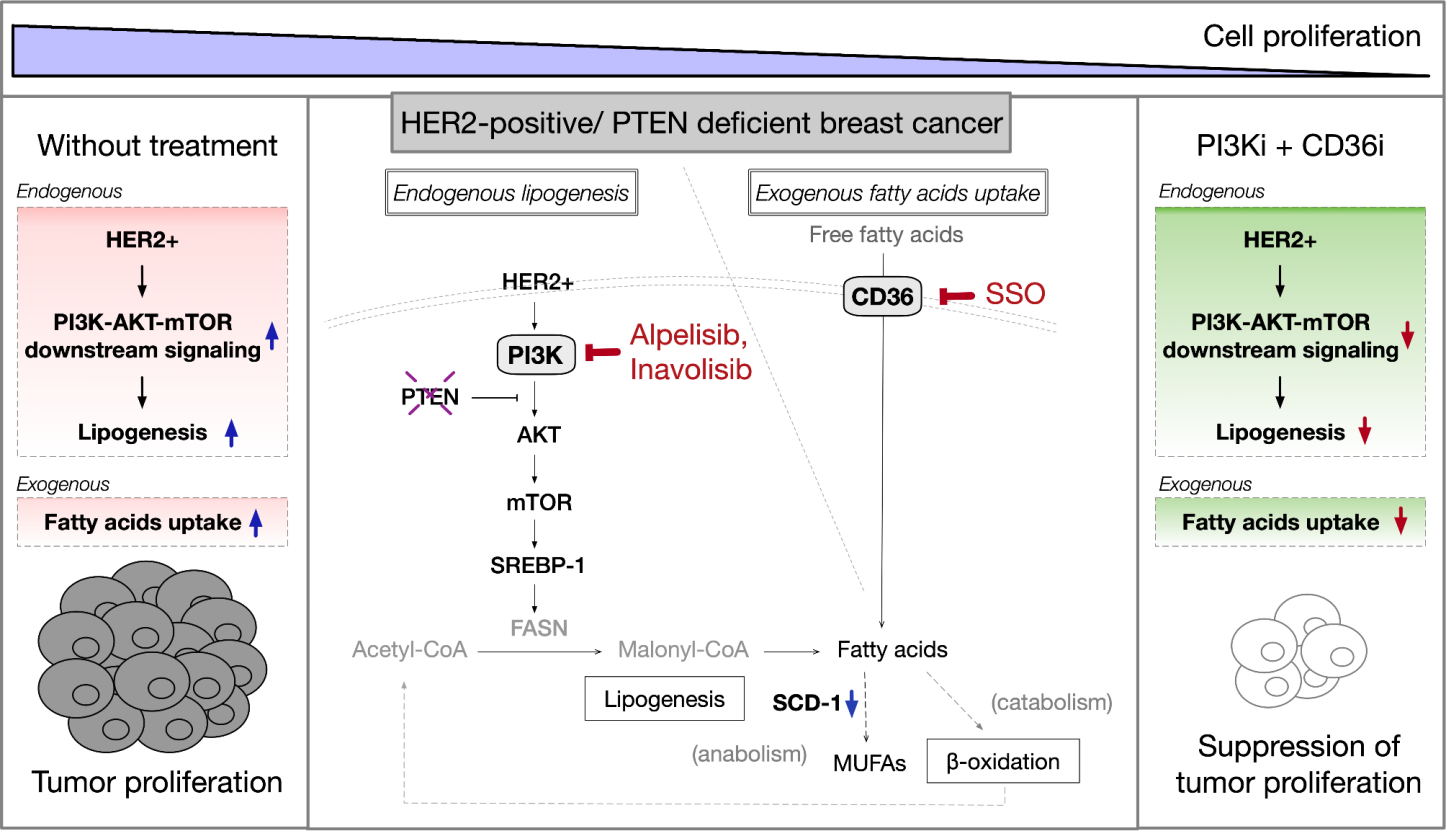
The combination strategy to suppress tumor cell proliferation in anti-HER2 resistant PTEN-deficient breast cancer cells. The constitutive activation of the HER2/PI3K/AKT/mTOR pathway led to the remodeling of lipid metabolism and cell proliferation in HER2-positive breast cancer cells with *PIK3CA* mutation or PTEN loss. SCD-1 and CD36 are highly expressed in anti-HER2 resistant breast cancer cells with PTEN loss. Simultaneously blocking the PI3K/AKT/mTOR signaling pathway and inhibiting CD36 significantly reduces the proliferation of anti-HER2 resistant breast cancer cells with PTEN-loss

The working model of this study was shown in Fig. 5. Inhibition of HER2/PI3K/AKT/mTOR signaling with PI3K inhibitors suppressed the expression of SCD-1 for endogenous FA lipogenesis, which was to a further extent in the presence of CD36 inhibitor. The combination therapy with CD36 inhibitor additionally reduced the activity for exogenous FAs uptake, thus, leading to significant reduction of cell proliferation in anti-HER2 resistant breast cancer with PTEN-loss.

In this study, the HER2-positive SK-BR-3 and HCC1569 cells displayed induction of lipogenesis associated proteins, SREBP1 and SCD-1 compared to the HER-2-negative MDA-MB-231 cells, with higher expression in the anti-HER2 resistant HCC1569 cells with constitutive activation of the PI3K signaling pathway due to the loss of PTEN. Furthermore, blocking PI3K signaling by alpelisib or inavolisib reduced SREBP1 and SCD-1 expression, suggesting that HER2/PI3K/AKT/mTOR signaling regulated the *de novo* lipogenesis machinery in HER2-positive breast cancer cells. However, SCD-1 inhibition by A939572 did not enhance the anti-proliferative effect of the PI3K inhibitors in anti-HER2 resistant breast cancer with PTEN-loss. This might be attributed to the shared axis between PI3K signaling and SCD-1 expression, that is, blocking the PI3K pathway by alpelisib or inavolisib also reduced SCD-1 which might already have reached its maximal inhibition.

Furthermore, SREBP1 is the major transcription factor that binds to the promoter region and regulates the gene expression of SCD-1. In addition to SREBP1, multiple transcription factors can regulate SCD-1, including peroxisome proliferator-activated receptor α, and liver X receptor, etc [35]. Thus, the SCD-1 expression might be modulated in the absence of significant SREBP1 changes, as shown in Fig. 1d, that the SCD-1 expression is decreased but SREBP1 is constant in HCC1569 cells. Additionally, the transcriptional regulations of SCD-1 include metabolic homeostasis and nutrient factors, such as glucose and saturated FA (SFA) induce, but polyunsaturated FA (PUFA) represses the gene expression [35]. Thus, the inhibition of SCD-1 which causes accumulation of SFA by the blockade of conversion to MUFA might be one of the reasons why SCD-1 expression was increased with A939572 treatment. On the other hand, CD36 is capable of transporting exogenous long-chain fatty acids into cells, including both saturated and unsaturated fatty acids, while the presence of double bonds enhances the interaction of fatty acids with CD36 and facilitates uptake of MUFA and PUFA over SFA [35, 36]. Therefore, treatment of SSO blocks CD36-mediated uptake of MUFA and PUFA, leading to the downregulation of SCD-1 (Fig. 4d and e). Furthermore, PUFAs can activate AMPK and PPARα signaling pathways which cause inhibition of SCD-1 at transcriptional levels [35].

With breast cancer patients, the high expression level of SCD-1 is associated with significantly shorter recurrence free survival [37]. Targeting SCD-1 by the novel oleanolic acid derivative ZQL-4c overcomes trastuzumab-resistance [38]. Notably, SCD-1 converts SFAs into unsaturated FAs to maintain their equilibrium, causing the tumor cells to survive. Therefore, SCD-1 inhibition with excess SFAs might serve as an alternative therapeutic strategy [39].

The adipocyte-enriched tumor microenvironment might play a crucial role in the exogenous FA uptake in breast cancer [40]. In a study of breast tumor samples, high CD36 expression positively correlated with adipocyte infiltration [41]. The overexpressed CD36 might contribute to the transport of free FAs into breast cancer cells from surrounding adipocytes to support tumor growth and cancer progression [21]. CD36 expression is induced in patients with HER2-positive breast cancer treated with anti-HER2 therapies and is associated with poor prognosis, whereas CD36 knockdown by siRNA promotes cell apoptosis and suppresses tumor growth [42]. Furthermore, CD36 in interaction with FABP4 that enhance the import of free fatty acids from adipocytes in the tumor microenvironment leads to activation of STAT3 signaling, metabolic reprogramming with a shift toward beta oxidation and promoting breast cancer progression [43]. CD36 can maintain lipid homeostasis via the modification with palmitoylation which might facilitate MUFA uptake, decrease saturation-lipotoxicity and contribute to high-fat-diet-driven metastasis in breast cancer mouse models [44]. Therefore, CD36 might serve as a potential target to treat resistant HER2-positive breast cancer. In this study, CD36 was highly expressed in anti-HER2 resistant HCC1569 breast cancer cells compared to anti-HER2 sensitive SK-BR-3 cells and the HER2-negative MDA-MB-231 cells. Blocking exogenous FAs uptake by serum deprivation or by CD36 inhibition reduced proliferation of HCC1569 cells. SSO treatment further enhanced the anti-proliferative effects of therapeutic PI3K inhibitors in anti-HER2 resistant breast cancer cells with PTEN-loss. Furthermore, PI3K/AKT signaling directly regulated glucose transporter 4 and glucose metabolic enzymes [45, 46], therefore, combined PI3K and CD36 inhibitors might restrict the energy supply for tumor growth by synergistically reducing exogenous FAs and glucose resources.

A series of drugs including monoclonal antibodies (e.g. trastuzumab and pertuzumab), tyrosine kinase inhibitors (e.g. lapatinib, neratinib, and tucatinib) and antibody-drug conjugates (e.g. trastuzumab emtansine and trastuzumab deruxtecan) have significantly improved cure rates in early-stage HER2-positive breast cancer patients, prolonging the survival of patients in advanced metastatic stages. Nevertheless, resistance against these anti-HER2 treatments is still a major issue [47]. Previously, we showed that the PI3K inhibitor/trastuzumab combination therapy is effective against anti-HER2 resistant breast cancer cells bearing *PIK3CA* mutations and/or losing PTEN expression by the synergistic blockage of downstream signaling and inducing apoptosis in cultivated cells and a xenograft mouse model [15]. However, the combination therapy was more effective in cancers bearing *PIK3CA* mutations than in cancers with PTEN loss [15]. Currently, combination therapy regimens are undergoing evaluation in the clinical trials ALPHABET (NCT05063786: alpelisib in combination with trastuzumab) and INAVO122 (NCT05894239: inavolisib in combination with trastuzumab-pertuzumab) [47–49].

In the previous study, CD36 was higher in ER-positive than ER-negative cells, and the high CD36 expression could activate ERα, ER-targeted genes and p-ERK1/2 to promote cell cycles as well as might contribute to tamoxifen-resistance [30]. Along this line, silencing CD36 decreases viability and migration with more potent effects on ER-positive than ER-negative cells, and further restored tamoxifen-mediated inhibition of cell growth in tamoxifen-resistant breast cancer. Therefore, the previous results exhibited that CD36 might participate in proliferation, migration and tamoxifen-inhibited growth of ER-positive breast cancer cells [30]. Notably, the probability of recurrence-free survival is higher in ER-positive patients with low CD36 than with high CD36 expression, such scenario is the same in HER2-positive patients [30]. In this study, we used the three ER-negative breast cancer cell lines in avoiding the confounding hormone status, with the results concluding that CD36 might play roles in anti-HER2 resistance while attenuating anti-proliferative effects of PI3K inhibitors in HER2-positive PTEN-loss cells. The results in present study and the previous study [30] are consistent and mutually support each other. Taken together, simultaneously targeting CD36 might be promising to benefit patients for either the treatment with anti-ER or with anti-HER2 therapy.

The present study demonstrated that simultaneously inhibiting HER2/PI3K/AKT/mTOR signaling which also subsequently suppressed endogenous FA lipogenesis and additional exogenous FAs uptake using a combination therapy of PI3K and CD36 inhibitors could significantly reduce the proliferation of anti-HER2 resistant breast cancer cells with PTEN-loss. Furthermore, a new generation CD36-targeting drug VT1021 has recently advanced to a late-stage clinical trial [50, 51]. In light of the increased availability of information, we hope that the combination approach involving a PI3K inhibitor and a CD36-targeting medication can be subjected to clinical trials in the future.

## Conclusion

The addition of a CD36 inhibitor might boost the anti-proliferation effects of PI3K therapeutics in anti-HER2 resistant, PTEN-loss breast cancer cells, thus providing promising insights for the development of combination regimens based on approved PI3K inhibitors with a tolerable toxicity profile.

## Electronic supplementary material

Below is the link to the electronic supplementary material.

Supplementary Material 1

## Data Availability

The datasets utilized and/or analyzed during the current study can be obtained from the corresponding author upon request.
