# Supplementary material for: CD36 inhibition enhances the anti-proliferative effects of PI3K inhibitors in PTEN-loss anti-HER2 resistant breast cancer cells
Source: Cancer Metab. 2025 Feb 7;13:6. doi: 10.1186/s40170-025-00375-5 (PMC11806886; doi:10.1186/s40170-025-00375-5)
Supplement: Supplementary file 1 — Supplementary Material 1 [file 40170_2025_375_MOESM1_ESM.docx]

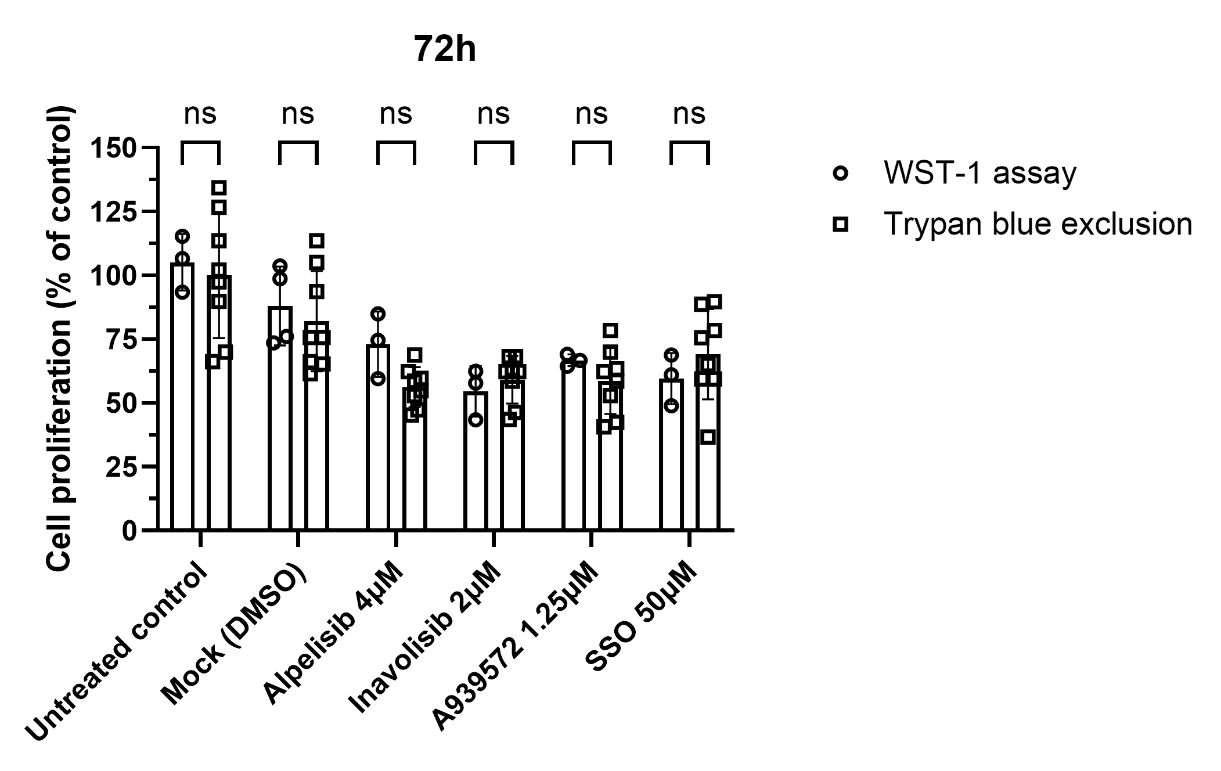
Figure S1. The anti-proliferative effects of HCC1569 cells treated with PI3K, SCD-1 and CD36 inhibitors were analyzed by WST-1 assay and trypan blue exclusion in parallel. The results (mean ± SD) exhibited similar inhibitory patterns by the two assays with the treatments of alpelisib (4 μM), inavolisib (2 μM), A939572 (1.25 μM), and SSO (50 μM) in HCC1569 cells at 72 hrs (p value > 0.05).


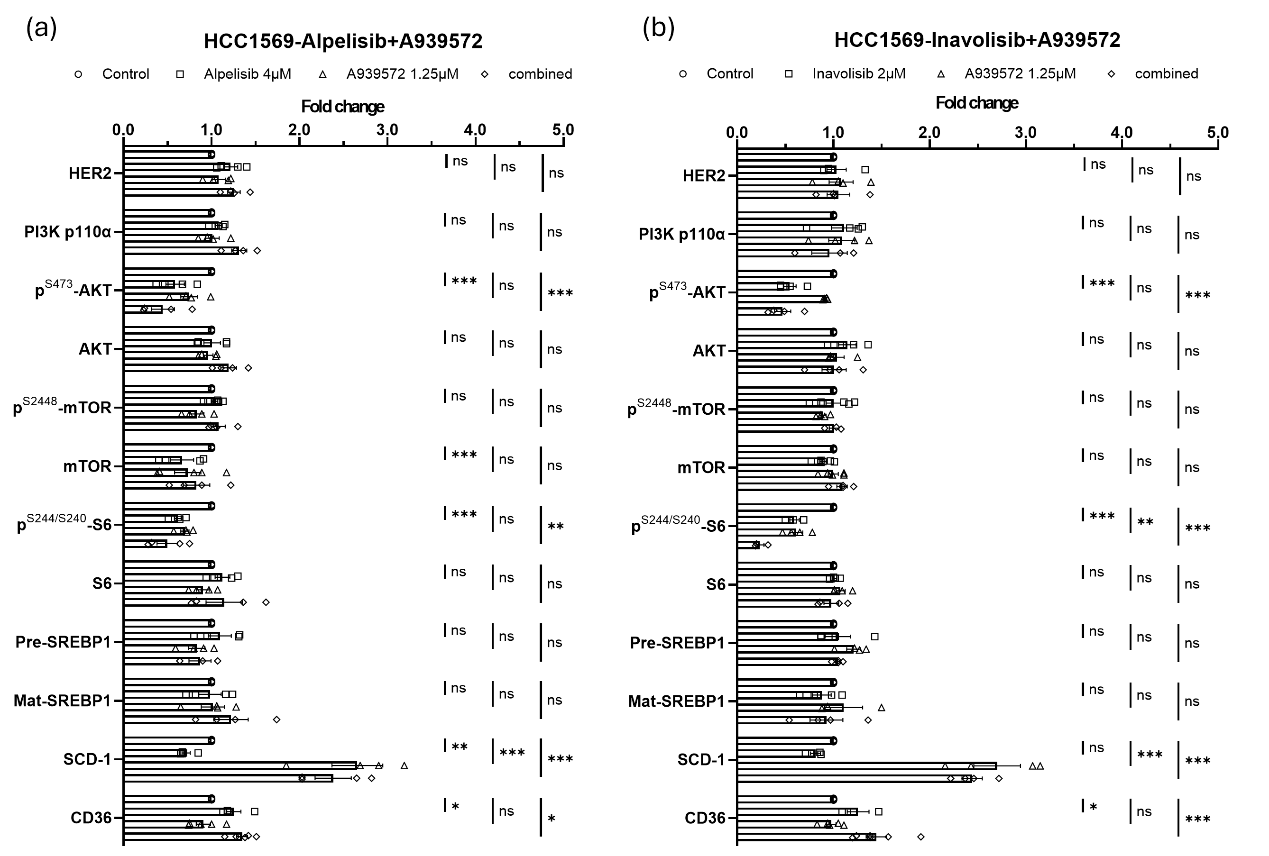


Figure S2. The corresponding graphs in which quantifying westerns by normalization to the same control treatment of (a) Fig. 2d and (b) Fig. 2e were presented. *P<0.05, **P<0.01 and ***P<0.001.


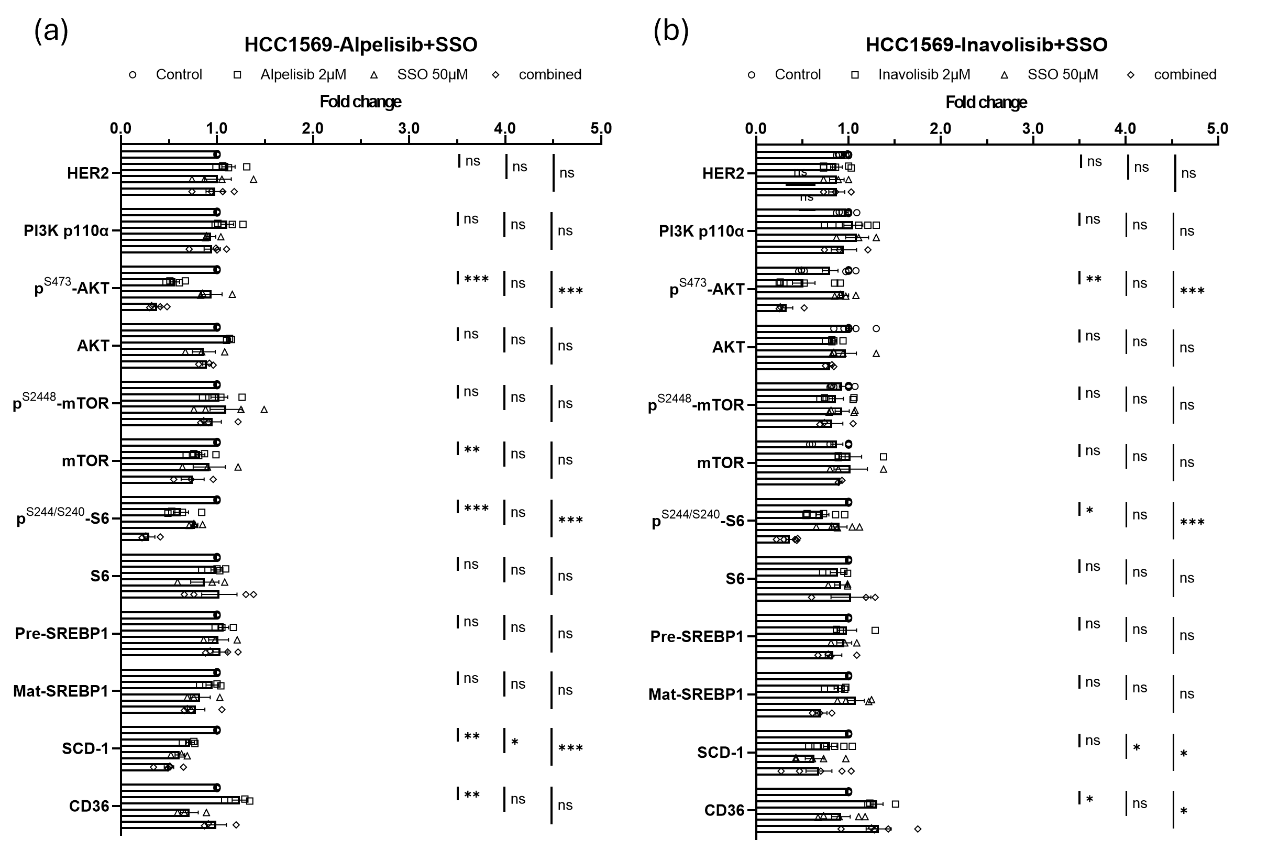


Figure S3. The corresponding graphs in which quantifying westerns by normalization to the same control treatment of (a) Fig. 4d and (b) Fig. 4e were presented. *P<0.05, **P<0.01 and ***P<0.001.


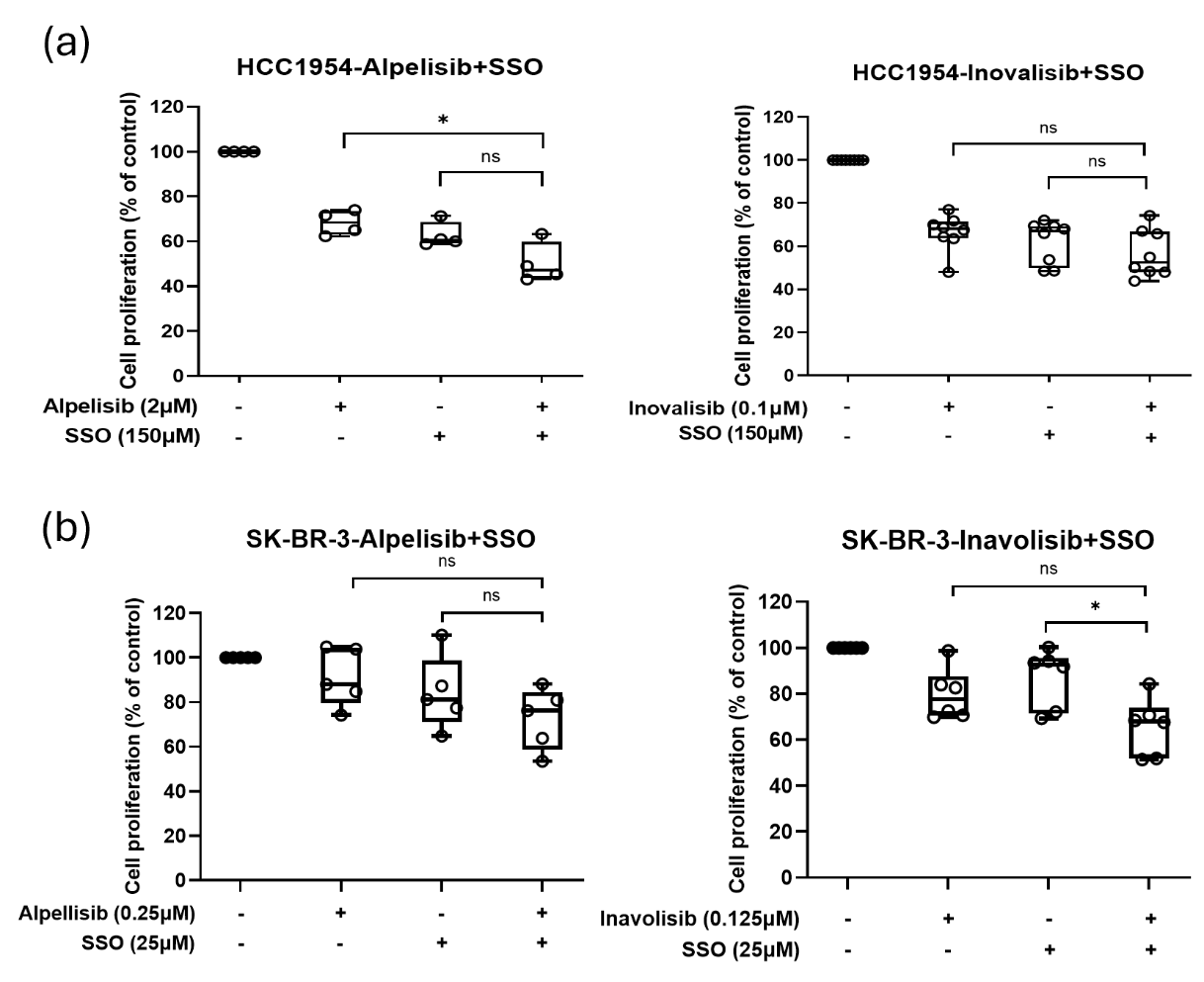


Figure S4. Co-treatment with SSO exhibited minimal enhanced anti-proliferative effects of alpelisib and inavolisib in 2 PTEN-positive cell lines, (a) HCC1954 (*PIK3CA* mutant) and (b) SK-BR-3 (*PIK3CA* wild-type)*.* Cell proliferation with the treatment of PI3K inhibitors plus SSO were assessed using the WST-1 assay and presented as a percentage of the untreated control by boxes and whiskers with minimum to maximum, *P<0.05, **P<0.01 and ***P<0.001.


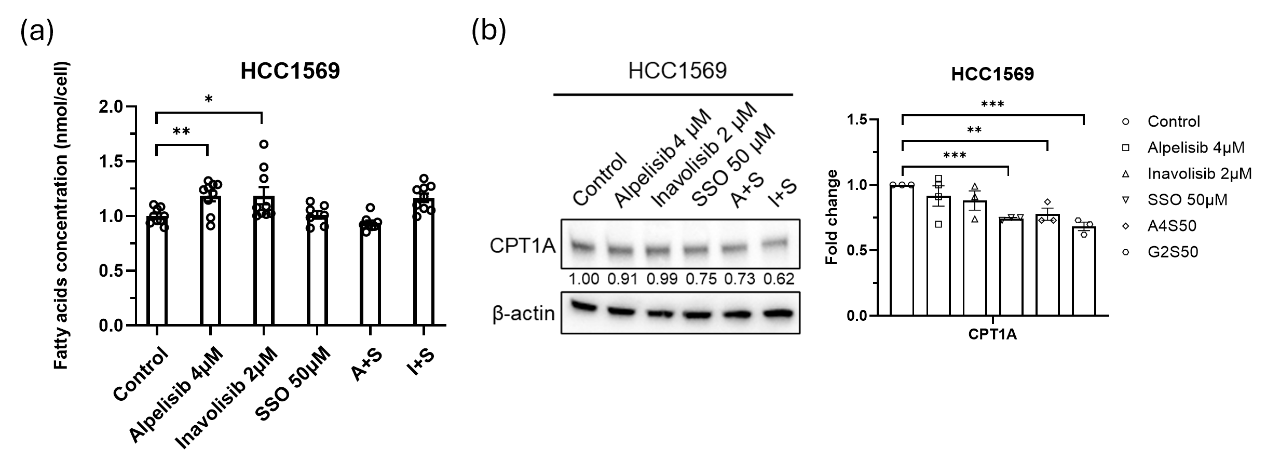
Figure S5. The effects on the pools of intracellular fatty acids and fatty acid beta-oxidation were evaluated. With the treatment for 24 hours, (a) the fatty acid levels were quantified using free fatty acid assay kit (ab65341, Abcam, UK) and (b) the expression levels of carnitine palmitoyltransferase 1A (CPT1A), the rate-limiting enzyme in mitochondrial fatty acid oxidation were detected using Western blot analysis (left) with quantification (right). *P<0.05, **P<0.01 and ***P<0.001.
